# Arm circumference predicts 12-month mortality in older adults with hip fracture

**DOI:** 10.1007/s41999-025-01364-z

**Published:** 2025-11-28

**Authors:** Chiara Ceolin, Giulia Termini, Stefania Sella, Valentina Camozzi, Anna Bertocco, Marco Onofrio Torres, Alberta Cecchinato, Martin Diogo, Mor Peleg Falb, Francesca Guidolin, Maria Grazia Rodà, Michele Cannito, Antonio Berizzi, Andrea Venturin, Vito Cianci, Elisa Pala, Mariachiara Cerchiaro, Deris Gianni Boemo, Maria Vittoria Nesoti, Gaetano Paride Arcidiacono, Paolo Simioni, Pietro Ruggieri, Giuseppe Sergi, Sandro Giannini, Marina De Rui, Hip-POS Working Group, Carlotta Andaloro, Gaetano Paride Arcidiacono, Giulia Bano, Antonio Berizzi, Anna Bertocco, Sara Bertolino, Deris Gianni Boemo, Ester Bukli, Valentina Camozzi, Davide Cannavò, Michele Cannito, Alberta Cecchinato, Chiara Ceolin, Mariachiara Cerchiaro, Vito Cianci, Giacomo Contini, Martina Dall’Agnol, Marina De Rui, Mario Degan, Marta Dianin, Martin Diogo, Michela Ferrarese, Claudia Finamoni, Sandro Giannini, Francesca Guidolin, Mario Rosario Lo Storto, Elena Marigo, Stefano Masiero, Caterina Mian, Maria Vittoria Nesoti, Elisa Pala, Mor Peleg Falb, Alessandra Pizziol, Maria Grazia Rodà, Giovanna Romanato, Paola Romano, Pietro Ruggieri, Cristina Russo, Sandro Savino, Stefania Sella, Giuseppe Sergi, Paolo Simioni, Cristina Simonato, Giulia Termini, Michele Tessarin, Marco Onofrio Torres, Andrea Venturin, Franz Villanova, Federica Vilona, Hillary Veronese, Francesca Zanchetta, Chiara Ziliotto

**Affiliations:** 1 Department of Medicine, University of Padua, Via Giustiniani 2, 35128 Padua, Italy; 2 Geriatrics Division, Department of Medicine, Azienda Ospedale-Università Padova, Padua, Italy; 3 Department of Neurobiology, Care Sciences and Society, Aging Research Center, Karolinska Institute and Stockholm University, Stockholm, Sweden; 4 Clinica Medica 1, Department of Medicine, Azienda Ospedale-Università Padova, Padua, Italy; 5 Endocrinology Unit, Department of Medicine, Azienda Ospedale-Università Padova, Padua, Italy; 6 Department of Orthopedics and Orthopedic Oncology, D.I.S.C.O.G. Department, University of Padua, Padua, Italy; 7 Orthopedics and Traumatology Unit, Azienda Ospedale-Università Padova, Padua, Italy; 8 Physical Medicine and Rehabilitation Unit, Azienda Ospedale-Università Padova, Padua, Italy; 9 Emergency Department, Azienda Ospedale-Università Padova, Padua, Italy; 10 Department of Directional Hospital Management, Azienda Ospedale-Università Padova, Padua, Italy

**Keywords:** Arm circumference, Handgrip strength, Fragility hip fractures, Fracture liaison service

## Abstract

**Purpose:**

Hip fractures (HF) in older adults are a major public health issue due to their high incidence and association with mortality and long-term disability. Simple bedside measures reflecting nutritional and muscular status—such as handgrip strength (HGS), arm circumference, and calf circumference—may help identify patients at higher risk of adverse outcomes. This study compared the predictive value of these parameters for 1-year mortality in older adults hospitalized for hip fracture.

**Methods:**

We conducted a retrospective study on 295 patients aged ≥65 years admitted for fragility HF at the Azienda Ospedale-Università Padova. Demographic, clinical, and anthropometric data were collected at admission, along with a comprehensive geriatric assessment. Twelve-month mortality was the primary outcome. Prognostic value was assessed using ROC curves, Kaplan–Meier analysis, and multivariable Cox regression.

**Results:**

At 12 months, mortality was higher among patients with low arm (27.1%) and calf circumference (28.8%) (*p* = 0.008 and *p* = 0.010), while no difference was observed for HGS. ROC curve analysis showed that arm (AUC = 0.704, *p* < 0.001) and calf circumference (AUC = 0.634, *p* = 0.006) were associated with mortality, unlike HGS (*p* = 0.307). Kaplan–Meier survival analysis confirmed lower survival in patients with arm and calf circumference under 20th percentile, defined, respectively, low arm and calf (log-rank *p* = 0.003 and *p* = 0.02, respectively). In Cox models, low arm circumference remained independently associated with mortality across all adjustments (HR = 2.86; 95% CI 1.45–5.64; *p* = 0.002), whereas the association for calf circumference lost significance.

**Conclusion:**

Arm circumference is a simple, cost-effective, and reliable bedside tool to help identify older adults at increased risk of mortality after HF.

## Key summary points

**Aim:**

To evaluate and compare the prognostic value of arm circumference, calf circumference, and handgrip strength for 1-year mortality in older adults hospitalized for hip fracture.

**Findings:**

Low arm and calf circumferences were significantly associated with increased 1-year mortality, while handgrip strength showed no predictive value. Arm circumference remained an independent predictor of mortality after full adjustment, whereas calf circumference did not.

**Message:**

Arm circumference is a simple, low-cost bedside tool that can reliably identify older hip fracture patients at increased risk of mortality.

## Introduction

Hip fractures represent a major global public health concern, given their high incidence and the substantial burden they impose [1–3]. Beyond their immediate clinical implications, these fractures significantly affect mortality rates, morbidity, functional independence, and quality of life in affected individuals [4, 5]. On a broader scale, they generate considerable costs for healthcare systems, primarily due to the long-term disability they often cause [6]. In older adults, the prognosis following a hip fracture is typically poor [7]. Functionally, the impact is severe, often resulting in increased rates of disability and the need for institutional care [4]. One-year mortality after a hip fracture in this population ranges between 15 and 36%, markedly exceeding that of the general population by threefold to fourfold [8].

Previous studies have examined predictors of short- and long-term mortality in patients with hip fractures [9–15]. According to a recent meta-analysis of 18 studies, several factors are significantly linked to increased mortality risk, including advanced age, male sex, cognitive decline, psychiatric conditions (such as delirium, dementia, and depression), dependence on a caregiver, multiple comorbidities, and the presence of cardiovascular, renal, or malignant diseases [9]. More recently, common geriatric syndromes like frailty and sarcopenia have also been recognized as important prognostic indicators [10, 11]. Beyond its role in the definition of sarcopenia, handgrip strength (HGS)—one of the simplest and most accessible measures of muscle strength, particularly in patients with hip fractures—has been linked to a wide range of clinical outcomes across different populations [16]. Notably, reduced HGS has been associated with increased mortality and disability in individuals with cardiovascular disease, those undergoing chronic hemodialysis, and patients with gastrointestinal cancers [11]. In the context of hip fractures, numerous studies have highlighted HGS as a valuable predictor of functional recovery following surgery, as well as of 1-year mortality in older adults [17–21].

Importantly, nutritional status is closely intertwined with sarcopenia, as malnutrition is both a contributor to and a consequence of declining muscle health [22]. In older adults with hip fractures, this bidirectional relationship plays a critical role in influencing clinical outcomes and rehabilitation potential [23]. Adequate nutrition supports key physiological processes—including bone healing, tissue regeneration, immune function, and wound repair—thereby reducing complications and promoting recovery [23]. Conversely, malnutrition—commonly observed in the older adults—can hinder the healing process, increase the risk of complications, delay functional recovery, and contribute to higher mortality and disability rates following a hip fracture [24, 25]. Given the critical role of nutrition in recovery, assessing and managing the nutritional status of older adults with hip fractures is an essential component of care [26]. Among the available tools, anthropometric measurements—such as arm and calf circumference—offer a simple, non-invasive, and cost-effective way to evaluate nutritional condition [23, 27, 28]. Low values in these parameters are frequently associated with malnutrition and have been linked to adverse clinical outcomes, including delayed wound healing, prolonged recovery, and reduced functional independence [29]. Beyond their value as markers of nutritional status, these measurements also provide meaningful insight into body composition. While arm circumference reflects subcutaneous fat and bone structure [30], calf circumference is particularly informative as an indirect measure of muscle mass. Since the lower limbs contain more than half of the body’s muscle mass—often compromised by immobility and disuse during illness—calf circumference is especially sensitive to changes in muscular health [30–32].

This study aims to evaluate and compare the prognostic value of two simple anthropometric indicators—arm and calf circumference—and a functional marker of muscle strength—HGS—in predicting 1-year mortality among older adults hospitalized for hip fracture. By evaluating and contrasting these anthropometric and functional parameters, the study seeks to identify accessible and reliable indicators that may assist clinicians in early risk stratification and individualized care planning in this high-risk population with hip fracture.

## Methods

### Study population

We conducted a retrospective study on patients aged 65 years and older, admitted with hip fractures at the Azienda Ospedale-Università Padova (Italy) within our Hip-POS FLS program. The complete organization of Hip-POS was previously described [6]. Briefly, we included only patients with fragility hip fractures assessed from March 2023 to March 2024, excluding those with traumatic (high-impact, non-fragility) or pathological fractures (e.g., primary or secondary bone tumors, Paget’s bone disease).

Written informed consent for the study was obtained at the patient’s initial clinical assessment. If a patient was unable to provide consent, caregivers/legal representatives were contacted by telephone and provided written informed consent on the patient’s behalf.

### Clinical data collection

For each patient, the following data were retrospectively retrieved from electronic hospital records, referring to the first 72 hof hospitalization:

- *Demographic and anthropometric characteristics*
- *Risk factors for skeletal fragility* (e.g., previous fragility fractures, family history of fractures, smoking habit, glucocorticoid use)
- *Laboratory tests*: These tests were conducted at the Laboratory Medicine Unit of the Azienda Ospedale-Università Padova, utilizing methods monitored for quality performance in accordance with the ISO 15189 standard. Lithium-heparin plasma samples were collected for calcium and phosphate measurements using a colorimetric method, and for creatinine via an enzymatic assay (calibrated to the reference procedure). Albumin was measured using an immunoturbidimetric method on the Cobas 8000 (Roche Diagnostics, Mannheim, Germany). Serum 25-OH-vitamin D and parathyroid hormone (PTH, third generation assay, reference range 6.5–36.8 ng/L) levels were measured using automated immunochemiluminescent methods (Liaison XL, DiaSorin, Saluggia, Italy) [6].
- *Multidimensional Prognostic Index *(*MPI*) [33]: The MPI is a prognostic index for 1-year mortality, calculated using information from the following scales (referred to the immediate period before the fracture): the Cumulative Illness Rating Scale (CIRS) for comorbidities [34], the Activities of Daily Living (ADL) [35] and Instrumental Activities of Daily Living (IADL) [36] for functional autonomy, the Mini Nutritional Assessment (MNA) [37] for nutritional status, the Short Portable Mental Status Questionnaire (SPMSQ) [38] for cognitive performance, and the Exton-Smith Scale (ESS) [39] for pressure sore risk. In addition, data on the patient’s medication regimen and cohabitation status were collected. The MPI score categorizes into three risk classes: class 1 (mild risk), class 2 (moderate risk), and class 3 (severe risk) [39].

As part of the HIP-POS program, muscle parameters were assessed within the first hours of admission, including the following measurements*:*

- *Anthropometry*. All measures were recorded at the admission of the patients, within the first 24–48 h, to exclude the worsened physical function due to hospitalization. Body weight was measured to the nearest 0.1 kg, using a standard balance with individuals wearing light clothes and no shoes; for those unable to walk, a lift scale was used. As most people were unable to maintain an upright position, body height was calculated from knee-to-heel length according to the Chumlea’s equations[40]. BMI was calculated as the ratio between weight (kg) and height squared (meters). Calf circumference was measured at the maximum circumference of the dominant calf [27, 41, 42], keeping the individuals in a supine position with the knee bent at 90°, using a measuring tape at the point of greatest diameter. An experienced physician checked for pitting edema before calf circumference measurement; furthermore, the measurements were all obtained in the morning to reduce the effect of edema. The feet were placed on the bed with the feet and ankles relaxed. Mid-upper arm circumference was measured on the dominant upper arm at the midpoint between the tip of the shoulder and the tip of the olecranon process [43–45].
- Muscle strength measurement: HGS was assessed using a calibrated electronic hand dynamometer (DynEx, Ohio, USA) by trained personnel. Testing followed a standardized protocol: participants were seated with the shoulder adducted and neutrally rotated, the elbow flexed at 90°, and the forearm and wrist in neutral; three maximal trials were performed for each hand with brief rest between trials. HGS for analysis was computed as the mean of the maximal values obtained from the dominant and non-dominant hands. Low muscle strength was defined according to EWGSOP2 sex-specific thresholds (<27 kg in men; <16 kg in women) [41, 46, 47].

After a 12-month follow-up, mortality data were collected through medical records or dedicated phone interviews with patients and/or their family members.

### Statistical analysis

The characteristics of the sample are expressed as means ± standard deviation for the continuous quantitative variables with a normal distribution, and as medians (interquartile range) for the variables with a non-normal distribution. The normality of the distributions of the continuous quantitative variables was assessed by the Shapiro–Wilk test. Categorical variables were expressed as counts and percentages. The characteristics of the study participants were compared with the Student’s *t* test for independent samples for parametric variables, the Wilcoxon rank sum test for non-parametric variables, and the Chi-square or Fisher’s test for categorical variables.

ROC curves were generated treating each predictor (arm circumference, calf circumference, HGS, and age) as a continuous variable, to evaluate their discriminative ability for 12-month mortality. We report the AUC with 95% confidence intervals and *p* values versus the null AUC = 0.5. For survival analyses (Kaplan–Meier and Cox regression), cut-offs were applied as follows: low HGS was defined according to sex-specific EWGSOP2 thresholds (<27 kg in men; <16 kg in women), while low arm circumference and low calf circumference were defined as values below the 20th percentile of the cohort distribution. These cut-offs were pre-specified and not derived from ROC analyses. Differences between groups at the Kaplan–Meier curves were assessed using the log-rank test. To further explore the association between these variables and mortality, Cox proportional hazards regression models were constructed. Hazard ratios (HRs) and 95% confidence intervals (CIs) were calculated. Three sequential models were developed: Model 1 adjusted for age and sex; Model 2 additionally adjusted for cognitive status, functional status, comorbidity burden, risk of pressure ulcers, length of hospital stay, and polypharmacy; and Model 3 further included nutritional status (MNA score). All analyses were performed on patients with complete data for handgrip strength, anthropometric measurements, and 1-year mortality; therefore, no missing data handling was required.

The statistical tests were considered significant at *p* < 0.05. All analyses were performed in IBM SPSS Statistics version 29.0 (IBM Corp., Armonk, NY, USA).

## Results

Table 1 presents the clinical and functional characteristics of the study population, stratified according to 12-month survival status. Among the 295 older adults included, 59 individuals (20%) had died at the 12-month follow-up. Patients who died were significantly older than survivors (mean age 86.7 ± 6.7 vs. 84.6 ± 6.9 years; *p* = 0.04), and a significantly lower proportion were female (52.5% vs. 76.3%; *p* < 0.001). In addition, length of hospital stay was notably longer among those who died (17.7 ± 10.8 vs. 13.5 ± 6.5 days; *p* = 0.001). Individuals who died within 12 months had significantly poorer profiles across functional, nutritional, cognitive, and comorbidity domains. Severe MPI scores were more frequent among deceased patients (23.7% vs. 11.4%), while mild MPI scores were more common among survivors (45.8% vs. 20.3%, *p* < 0.001). With respect to anthropometric and muscle strength assessments, both arm and calf circumferences were significantly lower among deceased patients (arm: 21.66 ± 9.37 vs. 25.00 ± 6.27 cm; *p* = 0.01; calf: 29.69 ± 4.66 vs. 31.15 ± 4.16 cm; *p* = 0.02). However, no significant difference was observed in HGS (*p* = 0.77). A greater proportion of deceased individuals fell below the 20th percentile for both arm circumference (27.1% vs. 12.3%; *p* = 0.008) and calf circumference (28.8% vs. 13.6%; *p* = 0.010). Although low HGS was highly prevalent in the overall sample, the difference between groups did not reach statistical significance (70.3% vs. 71.2%; *p* = 0.14). Regarding comorbidities, chronic obstructive pulmonary disease (COPD) was significantly more frequent among patients who died (15.3% vs. 5.1%; *p* = 0.02). Among osteoporosis-related variables (Table 2), a few noteworthy differences were observed between individuals who survived and those who died within 12 months. A family history of osteoporosis was significantly less prevalent among the deceased (6.8%) compared to survivors (18.2%; *p* = 0.03). Likewise, the use of vitamin D and calcium supplementation was more common among survivors (39.8%) than among those who died (23.7%; *p* = 0.02). No significant differences were found for other osteoporosis risk factors or for biochemical markers related to calcium-phosphate metabolism.

**Table 1:** Clinical, functional, and nutritional characteristics of the study population according to 12-month survival status

Variables	All sample (*n* = 295)	Alive at 12 months (*n* = 236)	Deceased at 12 months (*n* = 59)	*p* value
Age, years, means (SD)	85.0 (6.9)	84.6 (6.9)	86.7 (6.7)	0.04*
Sex, female *n* (%)	211 (71.5%)	180 (76.3%)	31 (52.5%)	<0.001***
BMI, kg/m^2a^, means (SD)	24.19 (4.05)	24.31 (4.11)	23.72 (3.80)	0.3
Length of stay, days, median (IQR)	14.44 (7.83)	13.52 (6.49)	17.68 (10.83)	0.001***
Multidimensional evaluation				
ADL^b^, means (SD)	4.51 (1.84)	4.67 (1.76)	3.88 (2.03)	<0.001***
IADL^c^, means (SD)	4.01 (3.08)	4.45 (3.03)	2.22 (2.61)	<0.001***
MNA^d^, means (SD)	21.22 (4.93)	21.84 (4.76)	18.74 (4.83)	<0.001***
ESS^e^, means (SD)	16.47 (3.22)	16.85 (3.0)	14.95 (3.58)	<0.001***
CIRS-CI^f^, means (SD)	3.51 (2.0)	3.30 (1.97)	4.32 (1.89)	<0.001***
SPMSQ^g^, means (SD)	3.45 (3.2)	3.09 (3.0)	4.92 (3.29)	<0.001***
Total no. drugs, means (SD)	4.67 (2.89)	4.58 (2.89)	5.03 (2.89)	0.3
Cohabitative status, *n* (%)				0.2
Family	182 (63.6%)	141 (61.3%)	41 (73.2%)	
Institutionalized	10 (3.5%)	8 (3.5%)	2 (3.6%)	
Alone	94 (32.9%)	81 (35.2%)	13 (23.2%)	
MPI^h^, *n* (%)				<0.001***
Mild	120 (40.7%)	108 (45.8%)	12 (20.3%)	
Moderate	134 (45.4%)	101 (42.8%)	33 (55.9%)	
Severe	41 (13.9%)	27 (11.4%)	14 (23.7%)	
Muscle assessment				
Arm circumference, cm^i^, means (SD)	24.33 (7.11)	25.00 (6.27)	21.66 (9.37)	0.01*
Calf circumference, cm^j^, means (SD)	30.86 (4.37)	31.15 (4.16)	29.69 (4.66)	0.02*
Handgrip strength, kg, means (SD)	15.33 (6.62)	15.17 (5.14)	15.52 (4.60)	0.8
Low arm circumference^k^, *n* (%)	45 (15.3%)	29 (12.3%)	16 (27.1%)	0.008**
Low calf circumference^k^, *n* (%)	49 (16.6%)	32 (13.6%)	17 (28.8%)	0.010*
Low handgrip strength^l^, *n* (%)	208 (70.5%)	166 (70.3%)	42 (71.2%)	0.14
Comorbidities, *n *(%)				
Hypertension	194 (65.8%)	156 (66.1%)	38 (64.4%)	0.9
Heart failure	19 (6.4%)	12 (5.1%)	7 (11.9%)	0.1
Ischemic heart disease	30 (10.2%)	23 (9.7%)	7 (11.9%)	0.6
COPD^m^	21 (7.1%)	12 (5.1%)	9 (15.3%)	0.02*
Diabetes	52 (17.6%)	39 (16.5%)	13 (22.0%)	0.3
Rheumatic disease	11 (3.7%)	10 (4.2%)	1 (1.7%)	0.7
GERD^n^/malabsorptive disorders	18 (6.1%)	14 (5.9%)	4 (6.8%)	0.8

^a^*BMI* Body mass index

^b^*ADL* Activities of Daily Living

^c^*IADL* Instrumental Activities of Daily Living

^d^*MNA* Mini Nutritional Assessment

^e^*ESS* Exton-Smith Scale

^f^*CIRS-CI* Cumulative Illness Rating **Scale—Comorbidity Index

^g^*SPMSQ* Short Portable Mental Status Questionnaire

^h^*MPI* Multidimensional Prognostic Index

^i^Arm circumference measured at the midpoint between acromion and olecranon

^j^Calf circumference measured at the maximum calf girth

^k^Defined as <20th percentile of the cohort distribution

^l^Defined according to sex-specific EWGSOP2 thresholds (<27 kg in men; <16 kg in women)

^m^*COPD* Chronic obstructive pulmonary disease

^n^*GERD* Gastroesophageal reflux disease

*p < 0.05; **p < 0.01; ***p < 0.001

**Table 2:** Comparative analysis of patients alive and deceased at 12 months, considering anamnesis of osteoporosis risk factors and calcium-phosphate metabolism

Variable	Alive at 12 months (*n* = 236)	Deceased at 12 months (*n* = 59)	*p* value
Osteoporosis risk factors, *n* (%)			
Family history of osteoporosis	43 (18.2%)	4 (6.8%)	0.03*
Early menopause	21 (11.7%)	2 (6.5%)	0.5
Previous major fractures (hip/vertebral)	44 (18.6%)	10 (16.9%)	0.7
Glucocorticoid therapy	3 (1.3%)	2 (3.4%)	0.3
Previous osteoporosis therapy, *n* (%)			
Vitamin D/calcium supplementation	94 (39.8%)	14 (23.7%)	0.02*
Bisphosphonates	17 (7.2%)	1 (1.7%)	0.1
Denosumab or Teriparatide	1 (0.4%)	0	1.0
Calcium-phosphate metabolism			
Calcium [mg/dL], mean (SD)	8.71 (0.52)	8.67 (0.64)	0.6
Phosphate [mg/dL], mean (SD)	2.92 (0.69)	2.87 (0.71)	0.6
PTH^a^ [ng/L], mean (SD)	42.39 (26.48)	45.79 (31.28)	0.4
Vitamin D [nmol/L], mean (SD)	46.44 (28.13)	38.80 (28.31)	0.1
Albumin [g/L], mean (SD)	28.96 (4.35)	27.86 (4.05)	0.1
Creatinine [mg/dL], mean (SD)	0.86 (0.35)	0.93 (0.45)	0.2

^a^*PTH* Parathyroid hormone

*p < 0.05

**Fig. 1:**
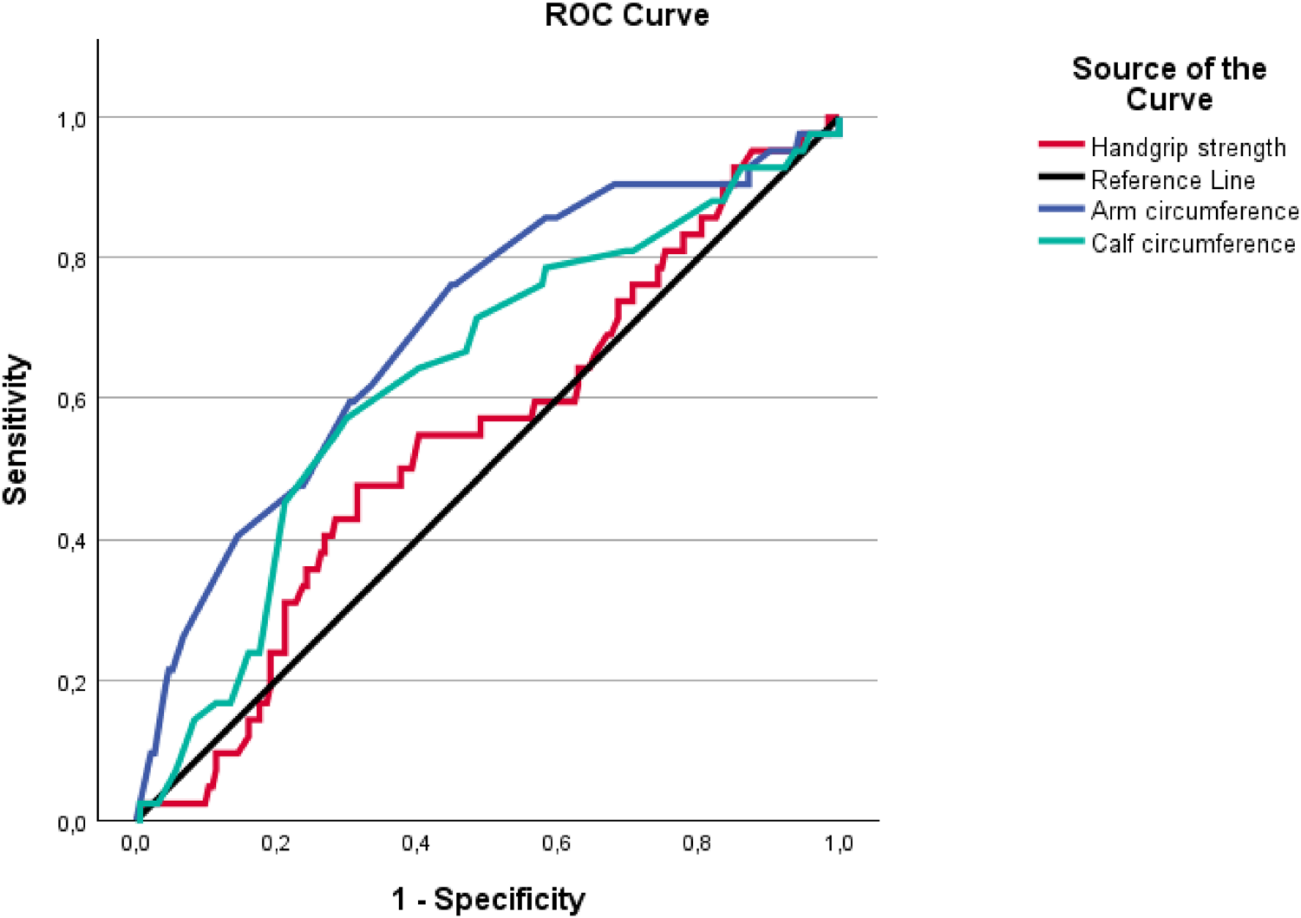
Comparison of arm circumference, calf circumference, and handgrip strength for predicting 12-month mortality: a ROC curve analysis

ROC curve analysis confirmed the prognostic value of arm and calf circumference (Fig. 1). Arm circumference showed a good discriminative ability for 12-month mortality, with an AUC of 0.704 (95% CI 0.616–0.791; *p* < 0.001), while calf circumference was also significantly associated with mortality, though with a lower AUC of 0.634 (95% CI 0.541–0.728;* p* = 0.006). HGS, however, did not show significant predictive value (AUC 0.550; 95% CI 0.455–0.645; *p* = 0.307).

**Table:** 

Test result variables	AUC	Std. error^a^	*p* value	Asymptotic 95% confidence interval		Comparison between curves
				Lower bound	Upper bound	
Arm circumference	0.704	0.045	** <0.001**	0.616	0.791	–
Handgripstrength test	0.550	0.048	0.307	0.455	0.645	<0.001
Calf circumference	0.634	0.048	**0.006**	0.541	0.728	<0.001

**Fig. 2:**
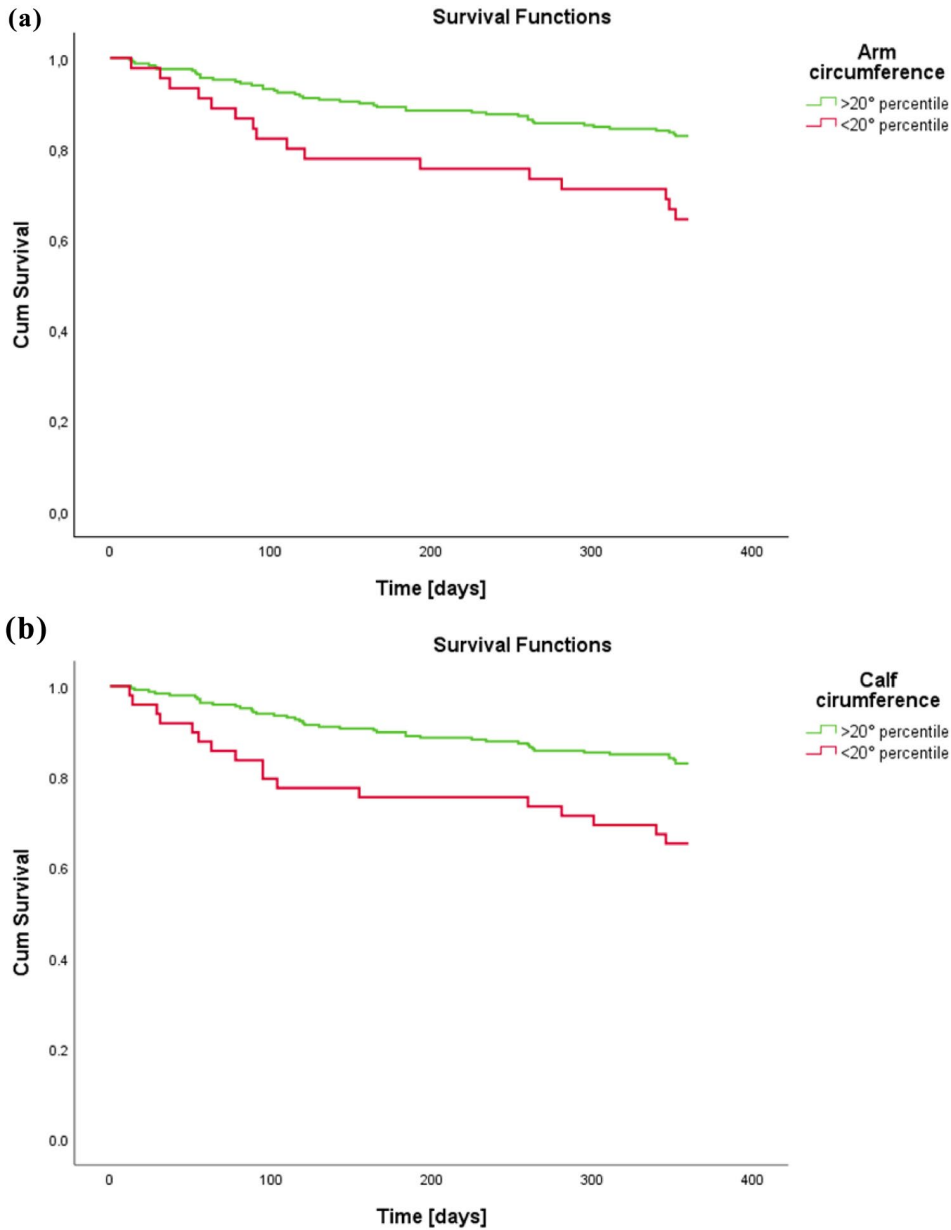
Kaplan–Meier curves stratified according to the 20th percentile of arm (**a**) and calf (**b**) circumferences

Based on these findings, further analyses were carried out for arm and calf circumference, stratifying patients according to the 20th percentile thresholds. Kaplan–Meier survival curves displayed in Fig. 2 demonstrated significantly reduced survival among individuals with low arm circumference (log-rank test, *p* = 0.003). A similar trend was observed for low calf circumference, which was also associated with decreased survival probability (*p* = 0.02).

In multivariable Cox regression models, low arm circumference remained a robust and independent predictor of 12-month mortality (Table 3). In the model adjusted for age and sex (Model 1), patients with arm circumference below the 20th percentile had nearly a threefold increased risk of death (HR 2.890; 95% CI 1.607–5.196; *p* < 0.001). This association persisted after adjustment for cognitive function, baseline functional status, comorbidity burden, pressure sore risk, length of hospital stay, and polypharmacy (Model 2: HR 2.931; 95% CI 1.495–5.748; *p* = 0.002), and remained significant even when nutritional status was included in the model (Model 3: HR 2.860; 95% CI 1.451–5.637; *p* = 0.002). Conversely, while low calf circumference was significantly associated with mortality in the age- and sex-adjusted model (HR 2.292; 95% CI 1.302–4.032; *p* = 0.004), this relationship was no longer statistically significant after adjustment for the broader set of clinical and functional covariates.

**Table 3:** Multivariable Cox regression analysis for 12-month mortality predictors

	Model 1^a^			Model 2^b^			Model 3^c^		
Variable	HR^d^	CI 95%	*p* value	HR^d^	CI 95%	*p* value	HR	CI 95%	*p* value
Low arm crf (<20° percentile)^e^	2.890	1.607;5.196	<0.001***	2.931	1.495;5.748	0.002**	2.860	1.451;5.637	0.002**
Low calf crf (<20° percentile)^e^	2.292	1.302;4.032	0.004**	1.528	0.822;2.840	0.180	1.462	0.781;2.737	0.236

^a^Model 1 adjusted for age and sex

^b^Model 2 adjusted for age, sex, cognitive function (SPMSQ), functional status (ADL and IADL), Exton-Smith Scale, comorbidity burden (CIRS-CI), length of stay, and total number of drugs

^c^Model 3 further adjusted for nutritional status (MNA score)

^d^Hazard ratio

^e^Low arm circumference and low calf circumference were defined as values below the 20th percentile of the cohort distribution

*p* < 0.05; **p < 0.01; ***p < 0.001

## Discussion

This study highlights the prognostic relevance of muscle health in older adults hospitalized for fragility hip fractures. Among the anthropometric parameters assessed, arm circumference—but not calf circumference—showed an independent association with 12-month mortality. These findings may inform early risk stratification following hip fracture, while warranting confirmation in prospective cohorts.

Our study contributes to the expanding body of literature demonstrating that peripheral indicators of muscle mass, such as arm and calf circumference, are valid predictors of mortality, as they also reflect nutritional status[27, 48, 49]. Muscle health and nutritional status are widely recognized as two sides of the same coin, both representing important risk factors for mortality [50]. The literature presents heterogeneous results regarding sarcopenia markers and their prognostic value for mortality in patients with hip fractures [11, 17, 51, 52]. Compared with previous cohorts reporting HGS as a predictor of mortality and functional recovery [18, 53, 54], our results differ in that HGS did not discriminate 12-month mortality. A key methodological difference is timing: many studies assessed HGS preoperatively, postoperatively, or during rehabilitation, whereas our measurements were obtained at admission in the acute phase. This difference, together with a pronounced floor effect (≈91% below EWGSOP2 thresholds), likely attenuated between-subject variability and may explain the weaker HGS signal observed here. By contrast, arm circumference retained discrimination and remained independently associated with mortality.

Furthermore, our findings suggest that arm circumference may serve as a marker of muscle mass loss in a context where strength decline is already significantly advanced, as seen in patients with hip fractures. It could be that arm circumference better reflects the remaining muscle mass prior to the fall, capturing a process of muscle wasting that may not yet be fully manifested through other clinical indicators. This parameter, in addition to representing lean mass, also correlates with subcutaneous adipose tissue thickness, which plays an active role in modulating systemic inflammation and the response to post-surgical metabolic stress [55]. Subcutaneous adipose tissue serves as a reservoir of free fatty acids and adipokines (e.g., adiponectin) with anti-inflammatory effects, whose depletion may exacerbate post-traumatic catabolism [56]. The significant association of arm circumference also persisted after adjustment for nutritional status, suggesting that it integrates information not fully captured by clinically manifest malnutrition. From a clinical standpoint, routine assessment of arm circumference at admission may provide a pragmatic, low-cost signal of limited physiological reserves. Pending external validation, this measure could complement malnutrition screening tools and functional assessments to help identify patients who may benefit from tailored nutritional and rehabilitation strategies. The prognostic value of arm circumference was further substantiated by the stratified analyses based on the 20th percentile cut-off. Patients falling below this threshold exhibited markedly reduced 12-month survival, with Kaplan–Meier curves showing a clear separation from those with higher values. Importantly, in Cox regression models, low arm circumference remained a robust and independent predictor of mortality, even after comprehensive adjustment for age, sex, comorbidity burden, cognitive and functional status, nutritional state, and hospital-related factors. The observed hazard ratio approached threefold increased risk of death, reinforcing the potential utility of this simple anthropometric parameter in early risk stratification.

Regarding calf circumference, our study initially found an association with 12-month survival, which diminished in the multivariate model. While values below the 20th percentile were initially associated with decreased survival and higher mortality in age- and sex-adjusted models, this association lost statistical significance when broader clinical covariates were included. This is in contrast with our previous study, that underlined the predictive value of calf circumference in predicting mortality in hospitalized older adults [27]. This loss of significance may be explained by the dynamics of the acute post-fracture phase, in which calf muscles, as anti-gravity muscles, are particularly susceptible to disuse atrophy, with rapid declines in mass and quality occurring within days of immobilization [57]. Consequently, calf circumference may reflect a systemic frailty already in place rather than pre-morbid muscle reserve. In other words, the rapid modifiability of this parameter limits its independent prognostic value in acute settings. By contrast, arm circumference appears more resistant to acute depletion, and its reduction may evolve over a longer time frame, making it a more stable indicator of the pre-trauma muscle–adipose condition [58].

A finding that appears to diverge from the existing literature concerns HGS. Different studies have underscored its predictive value for mortality [11, 17–20]. However, in our study, HGS did not prove to be a competitive predictor in mortality risk discrimination, as evidenced by ROC curves. This discrepancy could be attributed to the timing of measurement: performed in the acute post-fracture phase, it is affected by pain, immobilization, and inflammatory states characterized by elevated pro-inflammatory cytokines (e.g., IL-6, TNF-α), reducing its reliability as a marker of stable muscle reserve [51]. Building on this, we propose a conceptual distinction: anthropometric measurements, being less affected by acute neuromuscular variability, may represent more robust structural markers, whereas HGS retains value as a dynamic functional indicator in non-acute contexts. This framework underscores the importance of distinguishing between long-term physiological reserves and functional markers that can fluctuate due to transient conditions. This conceptual framework is hypothesis-generating and requires validation in prospective designs.

This study presents several strengths worth highlighting. First, it focuses on a high-risk, clinically relevant population—older adults hospitalized for fragility hip fractures—who often experience poor outcomes but are underrepresented in prognostic research. Second, it applies easily reproducible and low-cost bedside measures, such as arm and calf circumference, which enhances the translational potential of the findings in everyday clinical settings, especially in resource-limited contexts. Third, the robustness of the association between arm circumference and mortality after comprehensive multivariate adjustment supports its potential as a reliable prognostic indicator independent of known confounders such as age, comorbidities, and nutritional status. However, some limitations must be acknowledged. First, the observational nature of the study does not allow for causal inferences. The relatively limited number of events in our cohort may increase the risk of overfitting in the multivariable Cox models; however, covariates were selected a priori on the basis of clinical relevance, and results were consistent across simpler adjustment models. Second, despite standardized procedures (morning measurements and edema checks), anthropometric assessments may still be subject to misclassification and were not cross-validated against imaging-based body composition indices. Third, inflammatory and nutritional biomarkers were not systematically collected, limiting mechanistic interpretation. The single-center, retrospective design may constrain generalizability. Although we adjusted for several covariates, the possibility of residual confounding cannot be excluded. Finally, measurements were taken during the acute hospitalization phase, which may have introduced variability, particularly in functional parameters influenced by pain or inflammation.

In conclusion, our study strengthens the notion that, in geriatric-orthopedic care, simple anthropometric measures should not be viewed as secondary to sophisticated tools, but rather as cornerstones of a comprehensive assessment of physiological and nutritional reserves. Arm circumference emerges as an ideal bridge between the structural and functional dimensions of the human body, offering valuable and easily obtainable information with direct implications for clinical management and care pathways in older patients with fragility fractures.

## Data Availability

The datasets analyzed during the current study are available from the corresponding author on reasonable request.
